# Exploring the Potential of Epiregulin and Amphiregulin as Prognostic, Predictive, and Therapeutic Targets in Colorectal Cancer

**DOI:** 10.3390/onco4040019

**Published:** 2024-09-26

**Authors:** Cara Guernsey-Biddle, Peyton High, Kendra S. Carmon

**Affiliations:** 1 Center for Translational Cancer Research, The Brown Foundation Institute of Molecular Medicine, The University of Texas Health Science Center at Houston, Houston, TX 77030, USA.; 2 Graduate School of Biomedical Sciences, The University of Texas MD Anderson Cancer Center and UTHealth Houston, Houston, TX 77030, USA.

**Keywords:** epiregulin, amphiregulin, epidermal growth factor receptor, colorectal cancer, cetuximab, panitumumab, biomarkers, monoclonal antibodies, antibody-drug conjugates

## Abstract

The epidermal growth factor receptor (EGFR) plays a critical role in regulating essential cellular processes that are frequently hijacked to promote cancer. In colorectal cancer (CRC) in particular, the EGFR signaling pathway is frequently hyperactivated via receptor and/or ligand overexpression and downstream oncogenic mutations. Current EGFR-targeted therapies for metastatic CRC (mCRC) include the monoclonal antibodies (mAbs) cetuximab and panitumumab. However, intrinsic and acquired resistance to EGFR-targeted mAbs are commonly observed. Thus, additional biomarkers are necessary to better understand patient sensitivity to EGFR-targeted therapies. Furthermore, therapeutic targeting of alternative EGFR pathway components may serve as one mechanism to overcome EGFR-targeted mAb resistance. In this review, we discuss the mounting evidence supporting EGFR ligands epiregulin (EREG) and amphiregulin (AREG), which are overexpressed in CRC with potential key roles in tumor progression, as predictive biomarkers for EGFR-targeted therapy sensitivity as well as mediators of therapy resistance; though further studies are necessary to validate the prognostic roles and mechanisms by which these ligands contribute to resistance. Additionally, we review recent advances towards therapeutic targeting of EREG and AREG in cancer through the development and use of EREG- and AREG-targeted monoclonal antibodies (mAbs) as well as antibody-drug conjugates (ADCs). We conclude with a discussion on the roadblocks to clinical implementation of EREG and AREG as biomarkers as well as approaches to enhance efficacy of current EREG- and AREG-targeted strategies.

## Simple Summary

Colorectal cancer (CRC) is the second-leading cause of cancer-related deaths in the United States with a 14% five-year survival rate when metastasized. Current treatment options for metastatic CRC (mCRC) include combination chemotherapy regimens, immune checkpoint therapy, and monoclonal antibodies (mAbs), such as those targeting the epidermal growth factor receptor (EGFR). However, efficacy of these therapies is limited. EGFR-targeted mAbs extend survival only in a subset of mCRC patients (10–20%), in part due to drug resistance. Thus, additional biomarkers are required to identify responsive patients and to develop novel targeting strategies that will overcome resistance. This review aims to provide a comprehensive overview of EGFR ligands epiregulin (EREG) and amphiregulin (AREG) as prognostic, predictive, and therapeutic targets in CRC.

## 1. Introduction

Colorectal cancer (CRC) is the third most common cancer and second-leading cause of cancer-related deaths in the United States and worldwide [1]. In the United States, five-year survival rate is high (91%) when tumors are localized to the colon or rectum, which is most commonly treated by resection, but it decreases drastically to 14% at late stage with metastasis [2]. Notably, an estimated 15–30% of CRC patients initially present with metastases, and 20–50% of patients with localized tumors eventually develop metastases, most commonly in the liver [3]. Current treatment options for metastatic CRC (mCRC) include combination chemotherapy regimens (e.g. folinic acid, fluorouracil, and oxaliplatin, FOLFOX; folinic acid, fluorouracil, and irinotecan, FOLFIRI), immune checkpoint therapy, and monoclonal antibodies (mAbs), such as those targeting the epidermal growth factor receptor (EGFR). As a member of the human epidermal growth factor family of receptor tyrosine kinases (RTKs), EGFR plays a key role in regulating essential cell processes through ligand-stimulated receptor activation. As such, EGFR signaling is frequently dysregulated in CRC to promote cell proliferation and tumor growth via receptor and/or ligand overexpression as well as downstream oncogenic mutations. Thus, EGFR-targeted mAbs cetuximab and panitumumab are often used in combination with chemotherapy for first-line treatment of mCRC. However, the efficacy of EGFR blockade by EGFR-targeted mAbs is limited, extending survival only in a subset of mCRC patients (10–20%) in part due to drug resistance [4]. Constitutively activating downstream mutations (i.e., KRAS) have been identified as predictive biomarkers of EGFR-targeted mAb response, though resistance is still observed amongst *KRAS* wildtype (WT) mCRC patients. Thus, additional biomarkers are needed to identify responsive patients and to develop novel targeting strategies to overcome resistance. This review will begin with an overview of the EGFR pathway in CRC with a particular focus on EGFR ligands epiregulin (EREG) and amphiregulin (AREG), which are commonly overexpressed in CRC. We will then provide an updated assessment of the prognostic and predictive roles of EREG and AREG in mCRC and EGFR-targeted therapy followed by new potential roles in EGFR-targeted therapy resistance. Finally, we will conclude with recent progress made towards therapeutically targeting EREG and AREG in cancer with novel mAbs and ADCs.

### 1.1 EGFR Signaling

**Figure 1.**
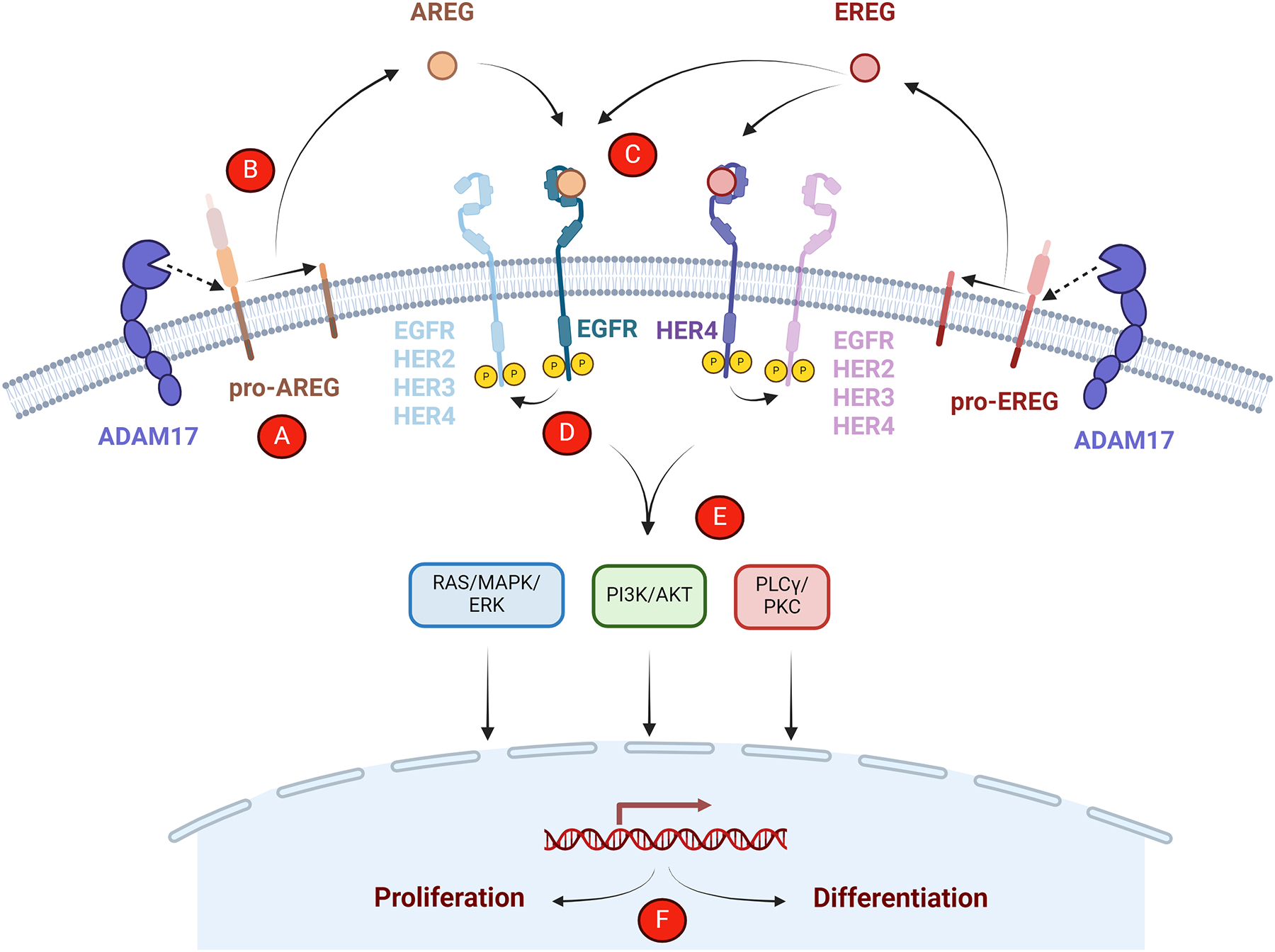
EREG/AREG processing and signaling through HER family receptors. EREG and AREG are synthesized as transmembrane pro-form proteins (A) cleaved by ADAM17 or other metalloproteinases (B) to release soluble growth factors that can bind their respective HER family receptor(s) (C). This induces receptor dimerization and autophosphorylation (D), after which adaptor proteins bind to potentiate downstream signaling (E). Furthermore, ligand identity is able to promote different cellular processes (e.g., proliferation and differentiation) (F).

The human epidermal growth factor family (ErbB/HER) of RTKs plays a critical role in regulating essential cellular processes, including proliferation, survival, differentiation, and migration among others [5]. The HER family is comprised of four transmembrane RTKs: the epidermal growth factor receptor (EGFR/ HER1/ ErbB1), HER2/ErbB2, HER3/ErbB3, and HER4/ErbB4. All HER family receptors share a similar structure with extracellular ligand-binding, transmembrane, and intracellular kinase domains [6]. EGFR, HER3, and HER4 require ligand binding to induce tyrosine kinase activation, receptor dimerization, and trans-autophosphorylation of the dimer partner [7]. In contrast, HER2 has no known ligand, constitutively exists in an active conformation, and is the preferred dimerization partner for all other HER family receptors [8]. There are eight HER family ligands, seven of which bind EGFR. Four of these seven have been shown to exclusively bind EGFR: AREG, epidermal growth factor (EGF), transforming growth factor-alpha (TGFα), and epigen (EPGN), while three bind both EGFR and HER4: EREG, heparin-binding EGF-like growth factor (HB-EGF), and betacellulin (BTC) [9]. Neuregulins (NRG1–4) bind HER4 and NRG1–2 bind HER3 [5]. As shown in Figure 1, all HER ligands are synthesized as transmembrane proteins that are then cleaved by metalloproteinases to produce a soluble growth factor capable of binding to its cognate receptor [10]. The ectodomain shedding of EGFR ligands is mediated by a disintegrin and metalloproteinases (ADAMs), primarily ADAM17/TACE and ADAM10, though other metalloproteinases may be involved [11]. Upon ligand binding and subsequent receptor autophosphorylation, Src homology 2 (SH2) and phosphotyrosine binding (PTB) domain-containing adaptor proteins bind phosphotyrosine residues in the C-terminal tail region to potentiate downstream signaling through RAS/MAPK/ERK, PI3K/AKT, PLCγ/PKC, and/or other pathways [12,13]. Importantly, ligand identity confers specificity for hetero- versus homodimer association and for particular phosphotyrosine residues, thus leading to binding of different adaptor proteins and the initiation of distinct signaling cascades depending on the activating ligand [14,15]. This may be due in part to ligand-specific stabilization of different dimeric receptor structures [16]. Further specificity is achieved by the strength and duration of ligand-induced dimerization as well as each ligand’s effect on receptor recycling versus degradation [16,17].

### 1.2. EGFR in Colorectal Cancer

Due to its central role in cell proliferation and survival, the HER signaling network is often hijacked to promote cancer pathogenesis. In CRC, HER signaling pathways may be dysregulated through both EGFR-dependent and EGFR-independent mechanisms. EGFR-dependent mechanisms include receptor overexpression, as EGFR amplification is observed in 65–75% of CRC patients with expression increasing throughout malignant transformation [18–20]. Additionally, EGFR ligand overexpression can promote CRC progression. For example, EREG [21] and AREG [22] are commonly overexpressed in CRC tumors with lower expression in normal tissues. In addition to receptor and ligand overexpression, downstream signaling mutations are commonly observed in CRC and confer independence from EGFR activation. For example, 40–45% of CRC patients harbor KRAS, 5–7% NRAS, and even fewer HRAS mutations while 7–15% harbor BRAF mutations, resulting in constitutive activation of the RAS/MAPK/ERK pathway [18]. Additionally, PIK3CA mutations are observed in 14–18% of mCRC patients [18].

### 1.3. EGFR-targeted Therapies

Due to the importance of EGFR signaling in CRC progression, EGFR-targeted mAbs are routinely used to treat mCRC [23]. Cetuximab (Erbitux^®^) was the first EGFR-targeted mAb approved for the treatment of KRAS WT mCRC in chemorefractory patients with panitumumab (Vectibix^®^) approval following shortly thereafter [6]. Cetuximab is a chimeric mouse/human IgG1 mAb, whereas pantimumab is a fully humanized IgG2 mAb [24]. Both mAbs bind EGFR at sites within extracellular domain III and compete with EGFR ligands for receptor binding to inhibit downstream signaling and cell proliferation. Additionally, these mAbs can bind Fcγ receptors to activate the immune system against cancer cells through antibody-dependent cellular cytotoxicity (ADCC) and antibody-dependent cellular phagocytosis (ADCP) [25]. However, the IgG1 composition of cetuximab promotes greater activation of Fc-mediated effector functions compared to panitumumab’s IgG2 composition.

The benefit of mAb-mediated EGFR blockade is largely limited to patients without constitutively activating downstream mutations (i.e. KRAS WT) [6,23]. Tumors with constitutively activating mutations downstream of EGFR no longer rely on ligand-stimulated EGFR activation to potentiate downstream signaling, thus rendering EGFR-targeted mAbs ineffective in inhibiting cell proliferation and tumor growth. Still, within KRAS WT patients, resistance to anti-EGFR mAbs is observed. Known mechanisms of resistance include alternative pathway activation (i.e. Hepatocyte growth factor receptor, MET), HER2 amplification, and microenvironmental plasticity among others [4]. Intriguingly, EREG and AREG have emerged as promising predictive biomarkers and novel therapeutic targets to overcome drug resistance to EGFR-targeted mAbs.

## 2. Epiregulin and Amphiregulin

EREG and AREG share similar gene regulation, structure, processing, and receptor signaling as EGFR ligands yet also potentially exhibit key functional differences as described herein.

### 2.1. Gene Regulation

In humans, the EREG and AREG genes are located on Chromosome 4, clustered among several other EGFR ligands [26]. Transcriptional regulators of EREG include insulin, specificity protein 1 (Sp1), activator protein 1 (AP-1), nuclear factor-κB (NF-kB), and the AP-2 family of transcription factors [27]. AREG has been shown to be transcriptionally regulated by many activators including Wilm’s tumor suppressor (WT1), hypoxia inducible factor-2 (HIF-2), p53, β-catenin, cAMP response element-bind protein (CREB), and AP1, while repressors include BRCA1 [28]. As with other EGFR ligands, EREG and AREG expression are both regulated via autocrine feedback loops [27,29–31]. Ligand-mediated EGFR activation and MAPK pathway signaling induce auto- and cross-expression of EGFR ligands that promote sustained signaling [29]. As such, EREG and AREG can regulate the expression of one another and are epigenetically co-regulated by DNA methylation [31–35]. EREG and AREG are also released and/or transcriptionally upregulated in response to G-protein coupled receptor (GPCR) agonists, such as lysophosphatidic acid, thrombin, and estradiol [36–38]. EGFR ligand secretion and subsequent EGFR transactivation in response to GPCRs is thought to be mediated, at least in part, by ADAM activation [39]. EREG and AREG expression can also be induced by cytokines and pathogen-associated molecular patterns, such as IL-1α, IL-1β, TNF-α, and LPS, indicative of their roles in inflammation and immunity [28,40,41]. While regulation of EREG and AREG expression have been shown in response to specific stimuli and mediated by specific transcription factors, the mechanisms by which regulation occurs are not entirely understood.

### 2.2. Protein Structure

Both EREG and AREG are synthesized as transmembrane proteins comprised of an extracellular N-terminal pro-region, EGF motif, and juxtamembrane stalk as well as the transmembrane domain and intracellular cytoplasmic tail [42]. Unlike EREG, though, AREG has an additional heparin-binding domain similar to HB-EGF. While their structure is highly conserved, EGFR ligand sequences lack homology, with only approximately 25% sequence conservation between all EGFR ligands [42]. These differences may contribute to receptor binding specificity.

### 2.3. Processing and Post-Translational Modifications

After synthesis, both EREG and AREG are glycosylated to produce approximately 30 kDa and 50 kDa cell surface pro-peptides, respectively, that can engage in juxtacrine EGFR signaling [43–45]. Notably, N-glycosylation mediates EREG stability by preventing ubiquitin-mediated proteasomal degradation [46]. Subsequent cleavage by metalloproteinases primarily releases 6 kDa and 43 kDa soluble forms of EREG and AREG respectively [44]. Alternative cleavage and processing can generate several other cellular and soluble forms of AREG, although this has not yet been demonstrated for EREG. ADAM17 is considered the primary sheddase for both AREG and EREG [47,48], although the role of ADAM17 in AREG shedding has been studied more extensively compared to EREG [49,50]. Furthermore, different stimuli, such as phorbol esters and calcium influx, can alter sheddase activity and stimulate alternative sheddases [48,51,52].

### 2.4. EREG/AREG-mediated Receptor Activation, Trafficking, and Downstream Signaling

Following cleavage, soluble EREG and AREG may activate EGFR in either an autocrine or paracrine manner. Upon binding to their respective receptors, EREG and AREG can induce trans-autophosphorylation of all four HER family receptors [53–55]. EREG and AREG are both low-affinity ligands that bind EGFR with 10 to 100-fold weaker affinity compared to EGF and TGFα, respectively [16]. It has been shown that EREG binding induces a distinct EGFR dimer configuration compared to TGFα, though the configuration induced by AREG has not yet been shown [16]. Compared to AREG, saturating levels of EREG have been shown to induce less EGFR homodimerization yet lead to more sustained C-tail phosphorylation and downstream signaling versus a more transient response, such as that mediated by AREG [16]. Both EREG and AREG have been shown to activate ERK and AKT [16,30,55], while AREG has also been shown to signal through PLCγ [15]. When propagated through ERK or AKT, sustained EREG signaling tends to promote differentiation whereas transient AREG signaling tends to promote proliferation [16,30,56,57]. Furthermore, EREG, among other EGFR ligands, has been implicated in the propagation of ERK activation waves during collective cell migration in MDCK cells, though AREG was not expressed [58]. EGFR-mediated ERK activity waves are also involved in maintaining epithelial homeostasis in response to injury and are associated with MAPK pathway amplification in KRAS and BRAF mutant CRC patient-derived organoids, potentially through EREG and/or AREG stimulated-activation [59,60]. Though, further work is necessary to resolve all EGFR-dependent and potentially EGFR-independent signaling pathways mediated by EREG and AREG.

After ligand binding, EGFR is internalized and trafficked to endosomes where it is then either degraded in lysosomes or recycled back to the cell surface. EREG and AREG both weakly induce EGFR internalization compared to other ligands, yet AREG-mediated stimulation internalizes more receptors compared to EREG at the same ligand concentration [17]. Both EREG and AREG primarily recycle EGFR back to the cell surface following internalization rather than promoting lysosomal degradation like other ligands, such as EGF, but EREG recycles EGFR more quickly compared to AREG [17]. Endocytic sorting of EGFR is, in part, affected by endosomal pH and receptor ubiquitination. Ligands that dissociate from EGFR at low pH tend to route EGFR for membrane recycling whereas ligand:receptor complexes that stay bound at low pH tend to favor receptor degradation. Intriguingly, AREG remains bound to EGFR at low pH despite promoting EGFR recycling rather than degradation [17]. All ligands also induce EGFR ubiquitination upon binding, but ligands that recycle EGFR tend to produce low (i.e. EREG) or transient (i.e. AREG) ubiquitination compared to strong and/or persistent ubiquitination that is associated with degradation [17]. Importantly, EGFR recycling allows for receptor re-activation and promotes sustained signaling. At the same time, EGFR ligand shedding also stimulates endocytosis of the transmembrane-cytoplasmic remnant stalk peptide, as well as unshed pro-peptides, which can be trafficked to the lysosome or inner nuclear membrane [11]. Nuclear trafficking of intracellular AREG peptides has been shown to regulate transcription epigenetically, though EREG and AREG do not stimulate EGFR nuclear translocation [61].

## 3. Prognostic and Predictive Roles of Epiregulin and Amphiregulin in CRC

EREG and AREG potentially play key roles in CRC progression. Both ligands are expressed at significantly higher levels in colorectal adenocarcinomas compared to early-stage adenomas [62]. This is due in part to decreased DNA methylation, as EREG and AREG expression are inversely associated with DNA methylation in CRC patients [34,62]. Accordingly, higher EREG and AREG expression are associated with low CpG island methylator phenotype (CIMP) status as well as WT KRAS/BRAF, left-primary tumor location (PTL), and microsatellite stability (MSS) versus microsatellite instability (MSI) [34]. However, EREG and AREG overexpression have still been observed across patient tumors regardless of KRAS BRAF mutational status and primary tumor location [63,64]. Furthermore, high EREG and AREG expression in primary tumors has been associated with metastasis [65], with their expression predictive of liver metastases in particular [66–68]. However, analysis of matched primary tumor and liver metastases showed EREG and AREG expression levels were not significantly different between the two tumor sites [67]. Furthermore, EREG and AREG expression are positively correlated in mCRC patients [64,67,69–74].

Despite their association with CRC progression and metastasis, studies on the prognostic effect of EREG and AREG expression on survival are limited by a lack of treatment naïve patients, such that biomarker-treatment interactions cannot be excluded. Studies conducted thus far are summarized in Table 1. When studied in RAS WT mCRC patients treated with EGFR-targeted therapy, high EREG and AREG expression were associated with improved survival regardless of the EGFR-targeted mAb treatment (i.e. cetuximab or panitumumab), combination chemotherapy therapy regimen (i.e. FOLFOX or FOLFIRI), and primary tumor location (i.e. left or right), even when adjusted for other prognostic variables, such as tumor grade and peritoneal metastases among others [64]. Similarly, high AREG expression was associated with survival benefit across treatment regimens in a pooled multi-trial analysis of mCRC patients treated with combination chemotherapy with or without cetuximab or the vascular endothelial growth factor A (VEGF-A)-targeted mAb bevacizumab [75]. However, with irinotecan-based chemotherapy treatment alone, EREG and AREG expression had no effect on survival in RAS WT and RAS mutant mCRC patients [73,74]. This suggests the impact of EREG and AREG expression on survival may be contextually dependent on a number of factors, including treatment regimen. Another study associated low EREG in primary tumors from KRAS WT mCRC patients with improved survival irrespective of EGFR-targeted therapy [67]. Additionally, high AREG and vascular invasion was associated with shorter disease- and hepatic metastasis-free survival in CRC patients without liver metastases at the time of resection [68]. However, additional patient cohorts should be assessed to validate these findings and better elucidate the prognostic roles of EREG and AREG throughout CRC progression.

EREG and AREG were identified as candidate biomarkers to predict EGFR-targeted therapy response as mRNA profiling of biopsies from mCRC patients treated with cetuximab showed differential EREG and AREG overexpression in responders with significantly longer progression free survival (PFS) [76]. As such, there have been several attempts since to validate EREG and AREG as biomarkers by retrospectively analyzing pretreatment specimens from metastatic or advanced CRC patients administered cetuximab or panitumumab with or without chemotherapy, as summarized in Table 1. When studied as separate biomarkers within KRAS WT patients, high EREG or AREG mRNA expression has been associated with improved survival and/or disease control when treated with cetuximab [63,69–71,74,75,77] and panitumumab [73]; however, depending on the analysis, the predictive effect of EREG may be more significant than AREG [63,74] or vice versa [73,78]. Interestingly, some multivariate analyses even suggested the predictive capability of EREG mRNA expression may be independent of KRAS/BRAF mutational status [69,79], while other analyses suggest KRAS mutant patients with high EREG or AREG expression still do not gain significant benefit from EGFR-targeted mAbs [63,69,70,73]. However, as EREG and AREG expression are highly correlated, multivariate analyses including the two as separate biomarkers can be affected by multicollinearity. Instead, a single dichotomized biomarker where patients with high EREG and high AREG are combined was found to be predictive of panitumumab response rate and survival benefit in KRAS WT patients independent of BRAF mutational status and primary tumor location [73,74,80]. When used to analyze EREG and AREG in RAS WT CRC patients receiving cetuximab or panitumumab therapy, the dichotomized model could predict significant effects on survival across primary tumor location, chemotherapy regimens, and EGFR-targeted mAbs [64]. In part, discrepancies in the predictive values of EREG and AREG may be explained by intrinsic differences in the clinical trials analyzed, including but not limited to patient sample size, type, population, and chemotherapy regimen. Despite discord on the utility of EREG versus AREG and the level of benefit gained, taken together, these studies demonstrate the importance of both EREG and AREG in predicting EGFR-targeted mAb response, particularly in KRAS WT patients. Thus, utilizing EREG/AREG expression as a combined biomarker may be the most conservative approach and useful strategy in selecting patients that will likely benefit from EGFR-targeted mAb therapy.

## 4. The Roles of Epiregulin and Amphiregulin in EGFR-targeted Therapy Resistance

**Figure 2.**
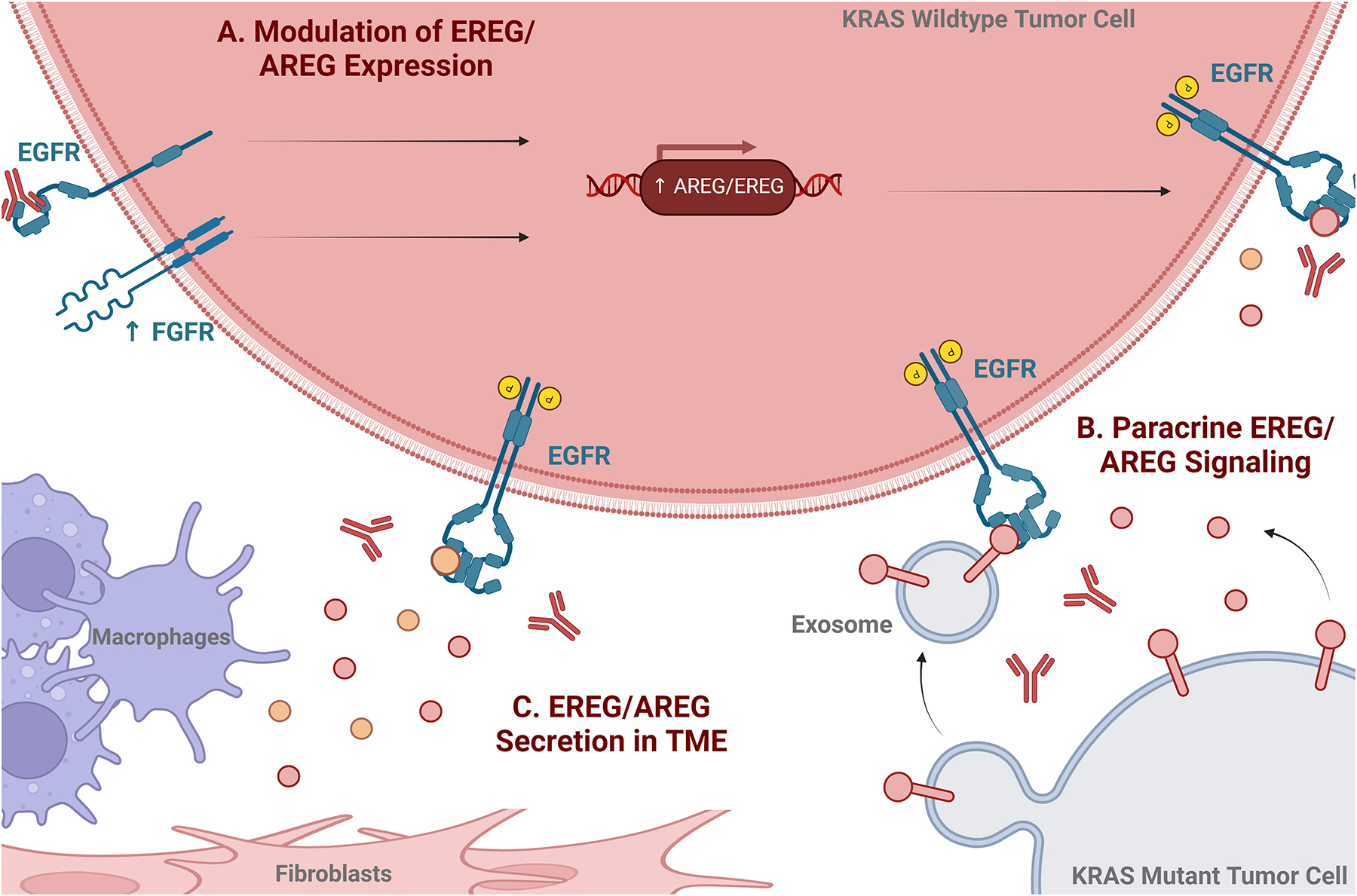
Potential mechanisms of EREG- and AREG-mediated EGFR-targeted mAb resistance. Mechanisms include ligand upregulation in KRAS WT tumor cells (A) as well as by paracrine ligand signaling from KRAS mutant cells (B) and soluble ligand secretion in the TME by cancer-associated fibroblasts and/or tumor associated macrophages (C), resulting in competition for EGFR-binding.

While EREG and AREG may play important roles in predicting EGFR-targeted mAb response, there is evidence that they may also contribute to therapy resistance through a variety of mechanisms as depicted in Figure 2.

### 4.1 Alterations in Ligand Expression

While EREG and AREG can predict response to EGFR-targeted mAbs, they may also act as mechanisms of resistance, in part through changes in their expression. A previous study showed that AREG and EREG expression can be modulated by fibroblast growth factor receptor 4 (FGFR4), an RTK known to promote proliferation, EMT, and decreased response to chemotherapy in CRC [81]. Overexpression of FGFR4 in CRC cell lines induced AREG and EREG mRNA expression, with a more significant increase in AREG at the protein level compared to EREG, amplified EGFR phosphorylation, and increased EGFR-HER3 heterodimerization. AREG siRNA decreased FGFR4-induced EGFR phosphorylation, suggesting the effect is, at least in part, ligand-mediated. Notably, overexpression of FGFR4 also reduced sensitivity to cetuximab *in vitro*. Furthermore, increased AREG secretion has been observed in RAS/BRAF WT CRC cells in response to cetuximab treatment, and treatment with recombinant AREG conferred cetuximab resistance in a cell line-dependent manner, though to a lesser extent compared to TGFα [82]. EREG secretion was not measured. These results suggest that AREG and/or EREG overexpression may confer acquired insensitivity to EGFR-targeted mAbs. However, EREG and AREG supplementation of cancer stem cell-enriched tumor spheroids from mCRC patient-derived xenografts did not confer protection from cetuximab treatment, while other EGFR ligands such as EGF and TGFα did [83]. Thus, while EGFR-targeted mAb resistance may be mediated in part by therapy-induced EREG/AREG upregulation and subsequent competition for EGFR binding, other EGFR ligands are likely important mediators as well.

### 4.2 Paracrine Growth Factor Signaling

Given that tumors are heterogenous, particularly when metastasized and subjected to several lines of drug pressure, tumor cells harboring specific oncogenic mutations (i.e. KRAS) may confer EGFR-targeted mAb resistance to otherwise treatment-sensitive WT cells via paracrine signaling [82,84]. In fact, conditioned media from CRC cells with acquired or conferred KRAS/BRAF mutations induced greater EGFR/ERK phosphorylation in the presence of cetuximab in WT CRC cells compared to conditioned media from parental cells [82]. Notably, the conditioned media from resistant cells contained increased levels of AREG and TGFα, suggesting that resistance may be mediated by paracrine growth factor signaling. However, treating WT CRC cells with recombinant AREG only conferred moderate protective effects at high concentrations compared to TGFα. While this could implicate TGFα or another soluble factor, such as EREG, as the primary mediator of cetuximab resistance, it is also possible that the effect is mediated by alternative sources of AREG. AREG, along with other EGFR ligands, has been detected in extracellular vesicles (EVs) secreted from CRC cell lines spanning different mutational statuses; though in KRAS mutant cell lines, AREG is enriched in EVs compared to cell lysates [85]. Secretion of AREG-containing EVs by KRAS/PIK3CA mutant CRC cells is also enhanced in response to stressors commonly found in the tumor microenvironment, such as nutrient deprivation and hypoxia [86,87]. Importantly, these EVs contain membrane-bound forms of AREG in a signaling-competent orientation [85,86]. Thus, AREG-containing EVs are able to enhance invasion and cell growth *in vitro*, including growth of KRAS WT CRC cells, and this effect is blocked by pre-incubation with anti-AREG antibodies [85,87]. Notably, these effects are more potent with AREG-containing EVs compared to soluble AREG, suggesting differential signaling and perhaps differential binding to EGFR [85–87]. As such, KRAS WT CRC cells were treated with cetuximab in the presence of stress-induced, AREG-containing EVs from KRAS mutant CRC cells [87]. Treatment with the EVs reduced cetuximab’s effect on cell growth, and addition of anti-AREG antibodies or EGFR/pan-HER receptor kinase inhibitors restored sensitivity. Taken together, this could suggest that membrane-bound AREG on EVs is better able to compete with cetuximab for EGFR binding compared to soluble AREG. Thus, is it possible that mutant (i.e. KRAS) tumor cells confer cetuximab resistance to WT tumor cells through paracrine signaling, perhaps in the form of AREG-containing EVs.

### 4.3 Growth Factor Secretion in the Tumor Microenvironment

Stromal remodeling, including increased infiltration of cancer associated fibroblast subtypes (CAFs), has been shown to mediate cetuximab resistance in CRC in the absence of acquired mutational resistance primarily via growth factor secretion [88]. Cetuximab sensitivity was shown to be attenuated when CRC cells are co-cultured with patient-derived CAFs and when incubated with conditioned media from cetuximab-treated CAFs [89]. Analysis of these CAF populations following cetuximab treatment *in vitro* showed upregulation of several cytokines and growth factors, including EGF, though addition of anti-EGF antibodies to CAF conditioned media only partially restored cetuximab sensitivity. While evidence on EREG and AREG secretion from CAFs in response to EGFR-targeted therapy is limited, it has been shown that EREG and AREG can be overexpressed and secreted from CAFs in colitis-associated CRC as well as other cancer types to promote tumor progression [90–92]. Furthermore, in a colonic adenoma double mutant mouse model, loss of adenoma polyposis coli (*Apc*) and indian hedgehog led to increased epithelial cell proliferation and EREG upregulation in colonic fibroblasts, while recombinant EREG was shown to promote proliferation of *Apc* mutant colonic organoids [93]. This further suggests that stromal cells may be an important source of EREG to support cell proliferation in CRC. Additionally, enhanced EREG expression in senescent stromal cells can be induced by DNA-damaging agents leading to pro-tumor effects and contributing to chemoresistance [94]. This raises the possibility that CAF-derived EREG and AREG could also play a role in EGFR-targeted mAb resistance, particularly when combined with chemotherapy [95]. However, EGFR-targeted mAbs can also bind CAFs. Upon direct treatment with cetuximab, CAFs appear to retain viability, although they may be killed by activated natural killer cells via cetuximab-mediated ADCC [89,96]. Thus, further research is necessary to determine the extent to which CAF-derived EREG and AREG play a role in mediating EGFR-targeted mAb resistance in mCRC patients.

Immune cells in the tumor microenvironment (TME) also play a key role in mediating EGFR-targeted therapy response. Cetuximab treatment has been shown to increase cytotoxic immune cell infiltrates in responsive mCRC patients, whereas nonresponsive patients lacked this induction [88]. However, the presence of tumor-promoting immune cells, such as tumor associated macrophages (TAMs) can complicate matters, in part by secreting growth factors in response to therapy [97]. While there is limited research on CRC TAM-secreted growth factors and EGFR mAb resistance, it has been suggested that macrophage-derived EREG may play a role in EGFR-targeted inhibitor resistance in non-small cell lung cancer (NSCLC) [97]. Single-cell analysis of patient samples revealed that EREG is primarily expressed by TAMs in the NSCLC TME and treatment with macrophage-conditioned media conferred resistance to small molecule EGFR inhibitor gefitinib in NSCLC cells. Furthermore, treatment of NSCLC cells with recombinant EREG conferred resistance to gefitinib by preventing apoptosis, and gefitinib treatment had no effect on EREG expression in TAMs. These results suggest that TAMs could be a potential source of EREG and/or AREG, which may confer resistance to EGFR-targeted therapies. These findings warrant further investigation into the role of TAM-secreted growth factors in response to EGFR-targeted therapies in mCRC.

## 5. Therapeutic Targeting of Epiregulin and Amphiregulin

Therapeutic targeting of EREG and AREG has been studied in the context of several different EREG and/or AREG-expressing cancer types. Current approaches of targeting EREG and AREG include mAbs and antibody-drug conjugates (ADCs) as summarized in Table 2.

### 5.1. Monoclonal Antibodies

AREG-targeted mAbs in preclinical development have shown promising anti-cancer effects in a variety of AREG-expressing cancer types, including ovarian, breast, and prostate cancer. In ovarian cancer models, AREG mAbs AR37 and AR30 inhibited tumor growth and/or prolonged survival [98,99]. Mechanistically, both mAbs targeted the extracellular EGF-domain, and neutralized AREG-mediated EGFR phosphorylation and downstream signaling. In vitro, AR37 blocked ligand-induced migration as well as incorporation of a radioactive nucleoside, indicating decreased DNA synthesis, though these effects were not studied with AR30 treatment [98]. In subcutaneous MLS cell line xenograft models, AR30 resulted in partial tumor growth inhibition (TGI) and synergized with cisplatin to significantly reduce tumor growth in part attributable to cisplatin-mediated AREG upregulation [99]. AREG mAb synergism with DNA-damaging agents was also observed in mice implanted with prostate or breast cancer cells together with AREG-expressing stromal cell counterparts, wherein an AREG mAb combined with mitoxantrone, reduced tumor volume more significantly than either monotherapy alone [100]. Importantly, mitoxantrone was shown to increase AREG expression and secretion in stromal cells. Notably, when mice were implanted with prostate cancer cells alone, AREG mAb combination therapy did not reduce tumor growth compared to mitoxantrone alone, suggesting that the improved efficacy of combination therapy relies on stroma-derived AREG. Since AR37 can bind both human and mouse AREG, the mAb was also shown to confer significant survival benefit and decreased ascites fluid volume in a syngeneic ovarian cancer model with no adverse effects [98]. Interestingly, this survival benefit was lost with AREG knockout suggesting that, in this model, AR37 efficacy depends on tumor-derived AREG rather than immune- or stromal-derived AREG. The conflicting roles of stromal-derived AREG in mediating mAb response may be due, in part, to varied tumor and stromal expression levels of AREG, with the latter potentially dependent on pretreatment with or without chemotherapy. Interestingly, treatment with AR37 also upregulated the chemokine ligand CXCL1, which is capable of activating EGFR and may be associated with an adaptive resistance mechanism to AREG-targeted mAbs.

Several mAbs have been developed to target EREG, specifically for the treatment of CRC. EREG-targeted mAb 9E5 recognizes Ser^26^–Phe^29^ in the pro-peptide region of EREG and showed nanomolar binding affinity to human EREG [102,103]. As expected, 9E5 neutralized EREG-stimulated EGFR phosphorylation and downstream signaling in CRC cells [104]. In vitro, 9E5 did not significantly affect CRC cell growth but impacted focal adhesion composition and decreased cell spreading [104]. Moreover, the humanized 9E5, referred to as HM1, was shown to mediate ADCC in vitro [102]. Similarly, treatment of CRC stem cells with another EREG-targeted mAb in vitro did not affect cell growth but induced ADCC [105]. EREG was shown to be expressed in both treatment naïve, leucine-rich repeat-containing, G protein-coupled receptor 5 (LGR5)-positive cells and LGR5+ cells that transitioned to drug-resistant LGR5-negative cells in response to chemotherapy [105]. As LGR5 is an important intestinal and cancer stem cell marker, this suggests EREG may be involved in cancer stemness. Administration of the EREG mAb following irinotecan treatment delayed growth of subcutaneous patient-derived xenografts enriched for LGR5 as well as decreased the number and size of metastatic tumor nodules after IV injection of the patient-derived LGR5+ CRC cells [105]. Similar to AREG-targeted mAbs, EREG-targeted mAbs were shown to synergize with mitoxantrone in mouse models implanted with prostate or breast cancer cells combined with stromal cells. The combination resulted in greater TGI compared to each therapy alone or cetuximab combined with or without mitoxantrone [94].

### 5.2. Antibody-Drug Conjugates

ADCs are a novel therapeutic modality of targeting cancer cells with mAbs conjugated to highly cytotoxic payloads that effectively act as target-guided missiles [106]. Upon binding their tumor-specific target antigen, ADCs are internalized and trafficked to lysosomes for payload release followed by tumor cell-killing, while sparing normal tissues. Depending on the payload, cancer cell apoptosis is commonly induced via DNA damage (e.g. duocarmycins, DuoDM), micro-tubule disruption (e.g. monomethyl auristatin E, MMAE), or topoisomerase I inhibition (e.g. camptothecin). ADCs are a rapidly growing and successful class of anti-cancer therapies, with 13 having received FDA approval and many more in clinical trials. Recently, ADCs directed against EREG and AREG have demonstrated therapeutic potential in preclinical development.

The first AREG-targeted ADC was developed by utilizing an AREG-targeted mAb 1A3 that specifically binds the neo-epitope of the cell surface residual stalk peptide generated following pro-AREG cleavage and release of the soluble AREG ligand [101]. This enables unique targeting of tumor cells that depend on proteolytic AREG cleavage. Importantly, binding of 1A3 to the AREG neo-epitope was shown to internalize and co-localize with lysosomes in breast cancer cell lines. To generate the ADC, GMF-1A3-MMAE, the 1A3 mAb was conjugated to a MMAE payload with a dipeptide cleavable linker via reduction-alkylation, with an approximate drug-antibody ratio (DAR) of 4. GMF-1A3-MMAE effectively killed breast cancer cell lines and impaired clonogenic growth in a dose-dependent manner. Furthermore, ADC treatment in orthotopic mammary xenografts resulted in partial or near complete regression; however, all mice experienced recurrence after 204 days. Thus, it would be interesting to evaluate GMF-1A3-MMAE efficacy in models of other AREG-expressing cancer types, including CRC.

Novel EREG ADCs were recently developed incorporating a humanized EREG-targeted mAb H231 that binds endogenous pro- and soluble EREG with high affinity [21]. H231 was shown to neutralize EREG-stimulated EGFR activation in CRC cell lines, internalize EREG independent of its receptors (i.e., EGFR/HER4), and colocalize with lysosomes. Site-specific chemoenzymatic conjugation of DuoDM using a branched dipeptide or tripeptide cleavable linker with or without β-glucuronidation resulted in the generation of EREG-targeted ADCs with a DAR of 4. All three ADCs tested in EREG-high CRC cells showed low nanomolar binding affinity and sub-nanomolar potency for cell-killing, though the tripeptide linker with DuoDM β-glucuronidation (H231-EGC-qDuoDM gluc) outperformed the other ADCs. This is likely due to enhanced hydrophilicity and stability associated with the tripeptide linker and β-glucuronidation [107,108]. All ADCs inhibited tumor growth in KRAS mutant EREG-high CRC cell line and patient-derived xenografts and were well tolerated, though the H231-EGC-qDuoDM gluc ADC showed increased tumor growth inhibition and survival.

## 6. Conclusion

EGFR signaling is an important pathway dysregulated in CRC to promote tumor progression and metastasis. However, inhibition of EGFR with FDA-approved EGFR-targeted mAbs is only effective in a subset of mCRC patients, indicating the need to identify new biomarkers for predicting response to therapy or the development of new targeted therapies. Interestingly, EGFR ligands EREG and AREG are observed to be highly upregulated in CRC and are close to being validated as a predictive dichotomized biomarker for EGFR-targeted mAb response, which will help identify patients likely to respond to EGFR-targeted mAbs independent of tumor mutational status and primary tumor location. However, further studies are needed to determine if KRAS mutant patients with right-sided tumors significantly benefit from EGFR-targeted mAbs when stratified by EREG/AREG expression. Yet despite overwhelming evidence that high EREG/AREG is an important predictor of EGFR-targeted mAb response, particularly in KRAS WT mCRC patients, they have yet to be put into clinical practice. Perhaps the biggest hurdle to clinical implementation is the uncertainty regarding dichotomization cut-off points to predict responders from non-responders. To address this, there is an ongoing ARIEL trial to evaluate response to chemotherapy with or without cetuximab or panitumumab in EREG/AREG high, RAS WT patients with right-sided tumors [109,110], using the combined EREG/AREG dichotomized model previously determined via retrospective analysis of the PICCOLO trial [73].

In addition to predicting EGFR-targeted mAb response, EREG and AREG may also mediate drug resistance through a variety of potential mechanisms, including ligand upregulation, paracrine growth factor signaling, and growth factor secretion by other cell types in the TME (i.e. CAFs, TAMs). Ultimately, increased EREG and AREG from any source may confer resistance by outcompeting EGFR-targeted mAbs for EGFR binding, thus re-activating EGFR and promoting tumor growth even in the presence of EGFR-targeted mAbs. However, further research is necessary to tease out the exact mechanism(s) by which EREG and AREG contribute to EGFR-targeted mAb resistance in CRC.

EREG and AREG are attractive therapeutic targets for CRC based on their high expression across tumors with different mutational statuses, lower expression in normal tissues, and potential roles in tumor progression [29,63]. MAbs and ADCs targeting EREG and AREG are in preclinical development and have thus far demonstrated to be well-tolerated while achieving TGI in variety of tumor models, including CRC. Combination treatment could be considered to enhance the efficacy of EREG- and AREG-targeted therapies, particularly in light of the synergy observed with chemotherapy in different studies. Modulation of EREG and AREG could also potentially be achieved by targeting regulators, such as ADAM17. However, despite its documented role in cancer progression, therapeutics targeting ADAM17 have been hindered by severe side effects due to its ubiquitous expression, wide variety of substrates, and catalytic domain conserved amongst other metalloproteinases [111]. Importantly, additional methods of addressing EGFR-targeted mAb resistance are being explored, including combination treatment with mAbs and small molecule inhibitors targeting alternative pathway activation such as MET (e.g. capmatinib), HER2 amplification (e.g. trastuzumab), and mutant KRAS/BRAF (e.g. sotorasib; encorafenib) among others [4,112]. Yet, EREG- and AREG-targeted therapies could eventually provide an alternative treatment strategy for CRC that is more effective and may benefit more patients compared to EGFR-targeted mAbs.

## Figures and Tables

**Table 1.** EREG and AREG as Prognostic and Predictive Biomarkers in CRC.

Biomarker Type	Patient Population	Treatment	Finding	Reference
Predictive	Advanced CRC	Cetuximab or best supportive care	High EREG associated with cetuximab benefit in KRAS WT patients	[63]
Prognostic/Predictive	RAS WT mCRC	Cetuximab or panitmumab +/− FOLFOX or FOLFIRI	High EREG/AREG associated with improved survival across treatment regimens	[64]
Prognostic	mCRC	Chemotherapy (fluoropyrimidine-based, none, or unknown)	Low EREG associated with improved survival in KRAS WT patients	[67]
Prognostic	CRC	N/A	High AREG/vascular invasion associated with shorter disease- and hepatic metastasis-free survival	[68]
Predictive	mCRC	Cetuximab	High AREG associated with benefit in KRAS WT; High EREG associated with benefit irrespective of KRAS mutational status	[69,79]
Predictive	mCRC	Cetuximab + irinotecan	High EREG/AREG associated with benefit in KRAS WT patients	[70]
Predictive	KRAS WT mCRC	Cetuximab	High EREG/AREG associated with benefit	[71]
Prognostic/Predictive	mCRC	Irinotecan +/− panitumumab	No association between EREG/AREG and survival with irinotecan alone High EREG/AREG associated with panitumumab benefit	[73,74,80]
Prognostic/Predictive	mCRC	Combination chemotherapy +/− cetuximab or bevacizumab	High AREG associated with survival benefit across treatment regimens High AREG associated with cetuximab benefit in KRAS WT patients	[75]
Predictive	mCRC	Cetuximab	High EREG/AREG associated with benefit	[76]
Predictive	mCRC	Cetuximab + irinotecan or oxaliplatin	High EREG/AREG associated with benefit	[77]
Predictive	KRAS WT mCRC	Anti-EGFR therapy + chemotherapy	High EREG/AREG associated with benefit	[78]

**Table 2.** EREG- and AREG-Targeted Therapies in Preclinical Development.

Agent Name	Target	Modality	Cancer Type	In Vivo Dosing	Safety	Reference
AR37	AREG	mAb	Ovarian	0.2 mg twice weekly	No effect on body weight or overall appearance	[98]
AR30	AREG	mAb	Ovarian	200 μg twice weekly	N/A	[99]
N/A	AREG	mAb	Prostate, Breast	10.0 mg/kg biweekly	No effect on body weight	[100]
GMF-1A3-MMAE	AREG	ADC	Breast	5 mg/kg every 4 days	No effect on body weight or overall appearance	[101]
9E5	EREG	mAb	CRC	N/A	N/A	[102–104]
HM1	EREG	mAb	CRC	N/A	N/A	[102]
N/A	EREG	mAb	CRC	N/A	N/A	[105]
N/A	EREG	mAb	Prostate, Breast	10.0 mg/kg biweekly	No effect on body weight, kidney and liver enzymes, or blood cell counts	[94]
H231-VC-cDuoDM	EREG	ADC	CRC	5–10 mg/kg weekly	No effect on body weight, kidney and liver enzymes, or blood cell counts	[21]
H231-EGC-cDuoDM						
H231-EGC-qDMDM gluc						
